# Platinum-based drugs for cancer therapy and anti-tumor strategies

**DOI:** 10.7150/thno.69424

**Published:** 2022-02-07

**Authors:** Chunyu Zhang, Chao Xu, Xueyun Gao, Qingqiang Yao

**Affiliations:** 1 Institute of Materia Medica, Shandong First Medical University & Shandong Academy of Medical Sciences, Jinan 250000, Shandong, China.; 2 Department of Life Science and Chemistry, Faculty of Environment and Life Science, Beijing University of Technology, Beijing 100124, China.; 3 College of Chemistry and Material Science, Shandong Agricultural University, Tai'an 271018, Shandong, China.

**Keywords:** Platinum-based drugs, Cancer therapy, Anti-cancer mechanism, Systemic toxicity, Platinum nanoclusters

## Abstract

Platinum-based drugs cisplatin, carboplatin, and oxaliplatin are widely used for chemotherapeutic eradication of cancer. However, the side effects of platinum drugs, such as lack of selectivity, high systemic toxicity, and drug resistance, seriously limit their clinical application. With advancements in nanotechnology and chemical synthesis, Pt-based anti-cancer drugs have made great progress in cancer therapy in recent years. Many strategies relied on the anti-cancer mechanism similar to cisplatin and achieved some success by modifying existing platinum drugs. Pt-based nanodrugs, such as platinum nanoclusters, have novel anti-cancer mechanisms and great potential in tumor-targeted therapy and have shown promising results in clinical application. In this review, we systematically explored the development of first-line platinum chemotherapy drugs in the clinic and their anti-cancer mechanisms. We also summarize the progress of Pt-based anti-cancer drug application in cancer therapy, emphasizing their modification to enhance the anti-tumor effect. Finally, we address challenges faced by platinum chemotherapy drugs, especially Pt nanocluster-based nanodrugs, in cancer treatment. The new platinum drugs and their targeted modifications undoubtedly provide a promising prospect for improving the current anti-cancer treatments.

## Introduction

Chemotherapy is an effective method of anti-tumor treatment 1-4. Before the 1960s, all drugs used to treat cancer were pure organic compounds 5. In the late 1960s, a simple coordination compound with anti-cancer properties, known as cisplatin, was accidentally discovered, and its cytostatic property to inhibit bacterial growth was detected. This discovery opened up a new possibility for cancer chemotherapy 6, 7.

The platinum-based anti-cancer drugs, including cisplatin 8, carboplatin 9, and oxaliplatin 10, with manifest therapeutic effects and well-defined mechanisms of action, are widely used in the clinic. As the first generation of the platinum anti-cancer drug, cisplatin has evident therapeutic effects on many malignant tumors, such as breast, ovarian, and colorectal 11, 12. However, cisplatin is a non-specific chemotherapeutic drug, causing systemic toxicity besides killing tumor cells 13. Thus, platinum anti-cancer drugs have serious undesirable effects, including dose-limiting toxicity, especially nephrotoxicity, neurotoxicity, ototoxicity, and myelosuppression 14, and long-term use of cisplatin causes serious damage to normal tissues 15. Due to the considerable therapeutic effect of cisplatin and other first-line clinical platinum drugs on tumor tissues, various strategies have been employed to reduce the damage to normal tissues, such as liposome encapsulation 16, 17, drug delivery by nanomaterial carriers 18, 19, and bioconjunction targeting highly expressed protein moieties on tumors 20, 21.

Many reformed and new anti-cancer platinum drugs have been formulated and reported recently. Among the diverse anti-cancer platinum drugs, multifunctional high-performance platinum nanocluster-based (Pt NC-based) nanodrugs, fabricated using different biocompatible materials leading to flexible designs, have attracted much attention for cancer-specific therapy and drug delivery 22, 23. Compared with traditional first-line clinical platinum drugs, Pt NC-based nanodrugs exhibited high stability, good water dispersibility and solubility *in vitro*, and low systemic toxicity and biocompatibility *in vivo* 24-26. The unique advantages of Pt NC-based nanodrugs for controllable fabrication, biosafety, and anti-tumor activity provide a broad prospect for their anti-cancer applications 27.

In this review, we summarize recent scientific advances in Pt-based drugs designed for anti-cancer treatment and address other relevant issues: (1) investigation of molecular mechanisms of platinum drugs in clinical use; (2) summary of the strategies developed to avoid systemic toxicity and improve bioavailability of platinum drugs; (3) development of novel Pt NC-based nanodrugs designed for anti-cancer treatment; (4) challenges of platinum chemotherapy drugs, especially Pt NC-based nanodrugs, for the anti-cancer treatment.

## Development of First-line Pt Drugs

Cisplatin, carboplatin, and oxaliplatin are clinically approved worldwide and are the first choice for malignant tumor treatment 28. As shown in Table 1, cisplatin is the first generation of Pt-based anti-cancer drugs, discovered in the late 1960s and approved for cancer treatment in 1978 29. Cisplatin has a therapeutic effect on many malignant tumors, such as breast, ovarian, and colorectal cancers. However, besides killing tumor cells, it is a non-specific therapeutic drug causing systemic toxicity 30 and serious damage to normal tissues by long-term use 31. Therefore, based on the first-generation platinum drug cisplatin, the second-generation platinum chemotherapy drug carboplatin was developed, which took more than 10 years to reach the clinic. Compared with cisplatin, carboplatin exhibits a lower hydration rate due to the bidentate cyclobutane dicarboxylic acid ligands 32, 33 and has high biosafety with greatly reduced systemic toxicity, including hepatotoxicity, nephrotoxicity, neurotoxicity, and ototoxicity 34. Because of its lower toxicity, carboplatin can be used as high-dose chemotherapy for aggressive tumors. Nevertheless, Pt drug resistance is the main concern in platinum chemotherapy 35. Cisplatin and carboplatin eventually produce drug resistance during treatment. Therefore, the third generation of platinum clinical drug oxaliplatin was developed. The mechanism of action of oxaliplatin is similar to cisplatin, without producing cross-resistance with cisplatin or carboplatin 36, 37. Therefore, oxaliplatin and cisplatin can achieve a complementary effect in clinical anti-cancer treatment and have been widely used 38-40. Although, much effort has been devoted to developing new platinum-based anti-cancer drugs, none has reached worldwide clinical application.

## Molecular Mechanism of Cisplatin

**Figure 1:**
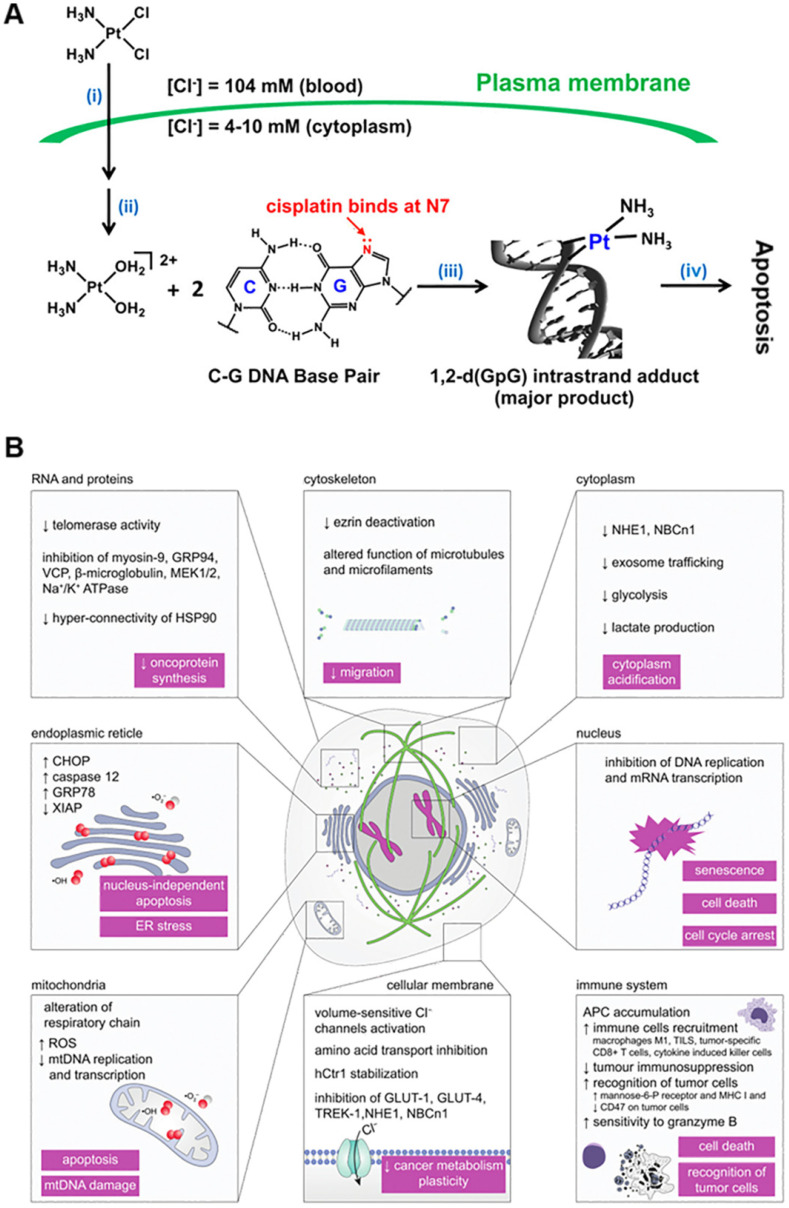
** Summary of the action mechanism of cisplatin. (A)** Mechanism of action of cisplatin comprising (i) cellular uptake, (ii) aquation/activation, (iii) DNA platination, and (iv) cellular processing leading to apoptosis. Adapted with permission from ref 44, copyright 2016 American Chemical Society. **(B)** Alternative effects of cisplatin. Other interesting mechanisms such as acidification of the cytoplasm, ER stress, disruption of RNA transcription, inhibition of important oncogenic proteins, and decrease in metabolic plasticity of cancer cells as well as changes in their mechanobiology. Adapted with permission from ref 46, copyright 2019 Royal Society of Chemistry.

As Pt-based anti-cancer treatment, cisplatin, carboplatin, oxaliplatin, and other drugs are widely used in the clinic with obvious therapeutic effects and a clear mechanism of action 41-43. Cisplatin is generally believed to be first transported into tumor cells through copper transporter 1 (CTR1). After entering the tumor cell, the platinum complex undergoes the activation step of chloro-ligand(s) replacement, generally by water molecules or other small molecules containing sulfhydryl groups. This replacement is triggered by the significantly lower intracellular chloride ion concentration (about 4 mM) compared to the extracellular matrix (about 100 mM), promoting transformation to cationic hydrate, such as cis-[Pt(NH_3_)_2_Cl(OH_2_)]^+^ and cis-[Pt(NH_3_)_2_(OH_2_)_2_]^2+^ 44. Due to the chelation of the leaving ligand, carboplatin and oxaliplatin are more stable to aquation. More importantly, the rate of hydration and reaction with ammonia for transplatin is much faster than cisplatin. Following a 4-h incubation with red blood cells, transplatin reacts with 70% of the glutathione, whereas cisplatin reacts with only 35% 45. The high reactivity of transplatin results in rapid deactivation of the complex before reaching its target, likely contributing to its lack of anti-cancer activity. After a series of chemical reactions in the cytoplasm, platinum binds to DNA by forming intra- and inter-stranded crosslinks, changing the DNA structure and causing DNA damage (Figure 1A) 46, 47. The most nucleophilic DNA site is the N7 position of guanine, which is exposed in the major groove and is preferentially platinated. This DNA damage can prevent the cell cycle and induce apoptosis in rapidly proliferating tumor cells 48, 49.

It is generally accepted that the principal mechanism of cisplatin anti-cancer action is platinum binding to DNA by forming intra-stranded and inter-stranded crosslinks 50, 51. However, some literature reported that probably only 1-10% of intracellular cisplatin might eventually enter the nucleus and react with DNA, resulting in cell cycle arrest and apoptosis in rapidly proliferating tumor cells. In this context, other novel action mechanisms, such as acidification of the cytoplasm 52, estrogen receptor (ER) stress 53, disruption of RNA transcription 54, inhibition of key oncogenic proteins, and decrease in metabolic plasticity of cancer cells and changes in their mechanobiology 55, have also been discovered (Figure 1B). The discovery of action mechanisms that may be affected by cisplatin may provide us with an important clue to design new anti-cancer treatment strategies by finding new potential therapeutic intervention targets.

## Strategies to Improve Anti-cancer Efficiency and Reduce Systemic Toxicity of Pt Drugs

Numerous studies have addressed the limitations of first-line platinum chemotherapy drugs due to potential toxicity and side effects and developed strategies to improve anti-cancer efficiency while reducing systemic toxicity 56. Abundant evidence has demonstrated that chemical modification of first-line platinum chemotherapy drugs to achieve targeted therapy is an effective method to effectively improve drug utilization and reduce side effects 16-18. In this section, we summarize these strategies, describing including bioconjunction targeting moiety, nanomaterials as drug carriers, and glutathione-scavenging Pt drugs.

### Bioconjunction Targeting Moiety

As stated earlier, the efficacy of first-line Pt drugs is limited due to the occurrence of severe side effects (nephrotoxicity, ototoxicity, peripheral neurotoxicity, and vomiting) together with the ability of cancer cells to limit drug accumulation 56. For Pt-based anti-cancer drugs, Pt(II) complexes are commonly used to treat malignant tumors. Photoactive Pt(IV) complexes are promising prodrug Pt(II) candidates activated by reduction in cancer cells and are being developed to lessen the side effects and improve pharmacological properties. Upon entering the cancer cells, the Pt(IV) center is reduced to Pt(II) and released 57. Under physiological conditions, the photosensitive Pt(IV) prodrugs retain their +4 valence state in the circulation system and are selectively converted to biologically active +2 valence state via mild ultraviolet light (UVA) irradiation after they reach the tumor 58, 59.

Extensive research indicated that the killing effect of Pt-based drugs on cancer cells could be improved by integrating the cancer cell-targeting moiety into Pt(IV) prodrugs. Notably, peptide-based drug delivery systems can enhance drug targeting properties and significantly reduce side effects 60, 61 due to their bioactivity and low immune response of peptides specifically expressed on tumor cell membranes 62-65. Peptide sequences with specific recognition characteristics of overexpressed proteins or other receptors can be introduced into Pt(IV) prodrugs to achieve the targeted function 66-68. More importantly, polypeptide sequences could be designed to perform different targeting functions. In recent years, our research group has made significant progress in functional targeted peptide design and exploring the biological effects of these peptides 69-71. In summary, different functional polypeptide-modified targeted platforms were developed to enhance effective utilization and reduce the side effects of Pt-based drugs.

**Figure 2:**
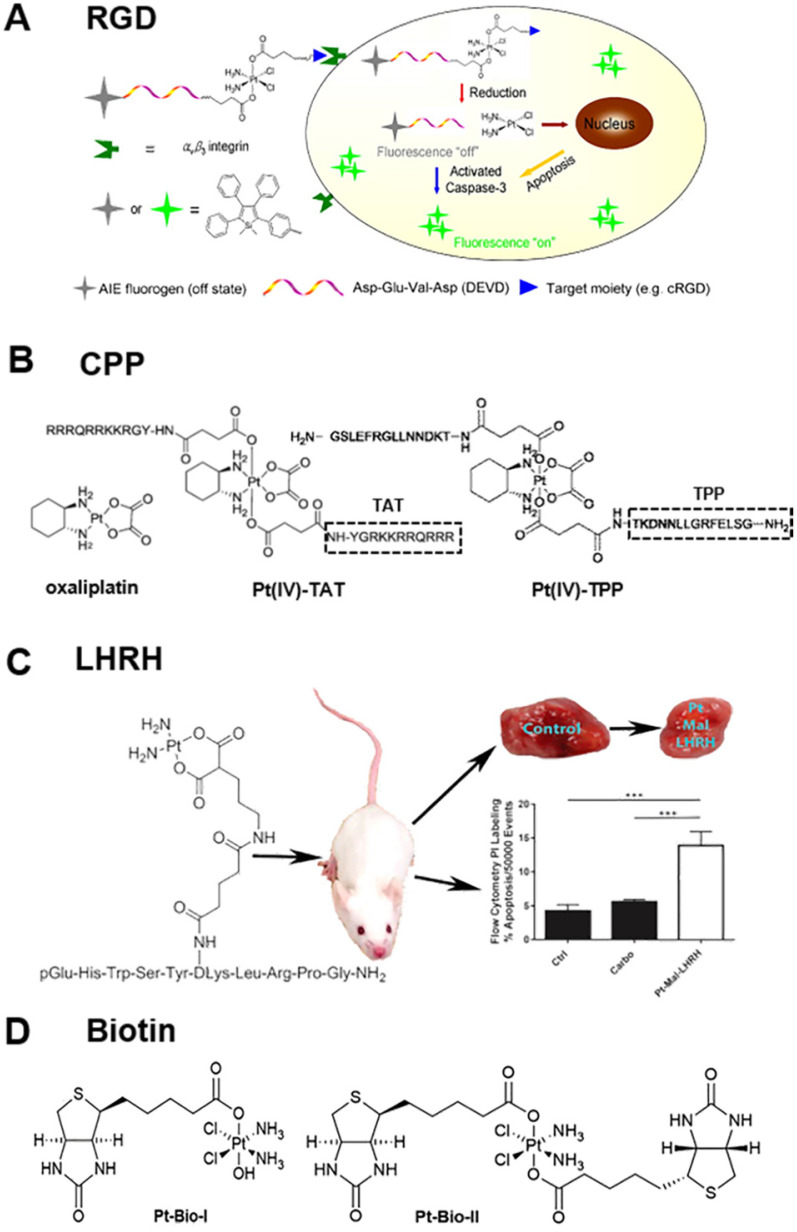
Summary of the bio-conjunction targeting moiety to improve the anti-cancer efficiency of Pt drugs, **(A)** RGD peptide 77, **(B)** CPP peptide 81, **(C)** LHRH peptide 9, and **(D)** biotin 84. Adapted with permission from ref 77, copyright 2014 American Chemical Society, ref 81, copyright 2017 Royal Society of Chemistry, ref 9, copyright 2017 American Chemical Society and ref 84, copyright 2017 Royal Society of Chemistry, respectively.

Integrins mediate cell-cell adhesion events and are overexpressed on tumor cell membranes with a key role in cancer progression 72, 73**.** The tripeptide arginine-glycine-aspartic (abbreviated as RGD) recognizes integrin α_v_β_3_ overexpressed on tumor cells 66, 74-76. Yuan et al. reported the synthesis and biological evaluation of a chemotherapeutic Pt(IV) prodrug whose two axial positions were functionalized with the cyclic tripeptide cRGD for targeting integrin α_v_β_3_-overexpressing cancer cells, an apoptosis sensor composed of tetraphenylsilole (TPS) fluorophore with aggregation-induced emission (AIE) characteristics, and a caspase-3 enzyme-specific Asp-Glu-Val-Asp (DEVD) peptide. The targeted Pt(IV) prodrug could selectively bind to integrin α_v_β_3_ overexpressed on cancer cells to facilitate cellular uptake. Furthermore, the Pt(IV) prodrug was reduced to active Pt(II) drug in cells, releasing the apoptosis sensor TPS-DEVD. The reduced Pt(II) drug could induce cell apoptosis and activate the caspase-3 enzyme to cleave the DEVD peptide sequence. Due to the free rotation of the phenylene rings, TPS-DEVD was nonemissive in aqueous media. The specific cleavage of DEVD by caspase-3 generated the hydrophobic TPS residue, which aggregated, resulting in restriction of intramolecular rotations of the phenyl rings and ultimately leading to fluorescence enhancement (Figure 2A) 77.

Similarly, Gandioso et al. reported an anti-cancer agent based on the conjugation of a photoactivatable Pt(IV) prodrug to a cyclic RGD-containing peptide 20. Upon visible light irradiation, phototoxicity was induced preferentially in SK-MEL-28 melanoma cancer cells overexpressing α_v_β_3_ integrin compared to control DU-145 human prostate carcinoma cells. The fact that the Pt-cyclo(RGDfK) conjugate (where f represents D-amino acids, and the others are L-amino acids) can also be internalized by α_v_β_5_ integrin opens up the door to delivering promising anti-cancer metallodrugs to tumors overexpressing α_v_β_5_ integrin or to tumors coexpressing α_v_β_3_ and α_v_β_5_ integrins. The multi-integrin targeting approach would provide new metal-based anti-cancer strategies and benefit a broader range of patients by increasing the types of tumors which can be targeted.

Furthermore, cell-penetrating peptides (CPP), such as HIV-1 TAT, which can penetrate proteins and oligoarginine, are valuable tools for transporting therapeutic macromolecules into tumor cells 78-80. As displayed in Figure 2B, McKeon et al. reported a novel Pt(IV) tumor-penetrating peptide (TPP) conjugate, which constitutes the first example of metallodrugs to target the membrane-bound heat shock protein 70-positive (memHSP70+) phenotype in cancer cells. The conjugates exhibited superior cytotoxicity as compared to oxaliplatin alone in Pt-resistant colorectal cancer cells with relatively high memHSP70+ expression. Substitution of TPP in Pt(IV) peptide conjugates with scrambled peptide (ScP) essentially abolished the observed cytotoxicity 81.

The luteinizing hormone-releasing hormone (LHRH) peptide also acts as a targeting moiety, whose receptors are overexpressed in several types of cancer cells, such as breast, ovarian, prostate, lung, and liver 9, 82, 83. The LHRH grafted with Pt drugs enabled selective accumulation and distribution of Pt drugs in tumor cells. Calderon et al. reported a new targeting chemotherapeutic agent, Pt-Mal-LHRH, synthesized by linking activated cisplatin to LHRH (Figure 2C) 9. They found that Pt-Mal-LHRH significantly enhanced cellular cytotoxicity in 4T1 cells compared to the normal 3T3 cell line. Both *in vitro* and *in vivo* data suggested that Pt-Mal-LHRH elicits tumor-targeted drug delivery with increased potency, efficacy, and a possible reduction in chemotherapeutic side effects allowing the use of a high dose of chemotherapy in patients compared to other platinum drugs. Also, *in vitro* scratch assay data demonstrated a reduction in migration of tumor cells. Importantly, *in vivo* metastasis was investigated since the major cause of mortality in breast cancer patients is metastasis to distant sites, including the lungs. The *in vivo* data supported the *in vitro* data, as a significant decrease was observed in tumor volume and lung tumor colonization by Pt-Mal-LHRH treatment. Thus, the Pt-Mal-LHRH conjugate was selective for tumors overexpressing LHRH receptors while avoiding systemic distribution.

Interestingly, conjugating non-functional small molecules with platinum drugs improves the targeting ability of Pt drugs. For example, Muhammad et al. reported the design and biological properties of two Pt(IV) complexes, Pt-Bio-I and Pt-Bio-II, carrying one or two biotin moieties in the axial positions of the Pt center (Figure 2D) 84. Tethering biotin moieties to the Pt(IV) prodrug remarkably increased cellular uptake of Pt in breast cancer cells but lowered its accumulation in breast epithelial cells. The mono-biotinylated Pt(IV) complex was more active than the di-biotinylated one in reactivity and cytotoxicity. Compared with cisplatin, Pt-Bio-I showed much stronger inhibition against cisplatin-insensitive MDA-MB-231 and MCF-7 cancer cells. Considering its low toxicity towards mammary epithelial cells, Pt-Bio-I may be superior over cisplatin in breast cancer therapy. Interestingly, Pt(IV) complexes with one hydroxyl ligand in the axial position appear to be beneficial for their interaction with DNA and cytotoxicity to cancer cells.

### Nanomaterials as Drug Carriers

#### Gold Nanoclusters (GNCs) as Drug Carriers

Nanocarrier-based platinum drug delivery systems are promising alternatives to avoid the disadvantages of conventional platinum drugs 22, 85, 86. In recent years, there has been much interest in GNCs as drug transport carriers due to their high photostability 87, water solubility, and biocompatibility 25, 88. Compared with traditional nanoparticles, the particle size of GNCs is usually less than 2 nm with increased blood circulation time. Pt drugs can be loaded efficiently in GNCs with increased accumulation in tumor tissues through the enhanced permeation and retention (EPR) effect, resulting in improved therapeutic efficacy and reduced systemic toxicity. Furthermore, ultrasmall GNCs are usually filtered out of the body through effective renal clearance, indicating good metabolism and biocompatibility 89-91.

**Figure 3:**
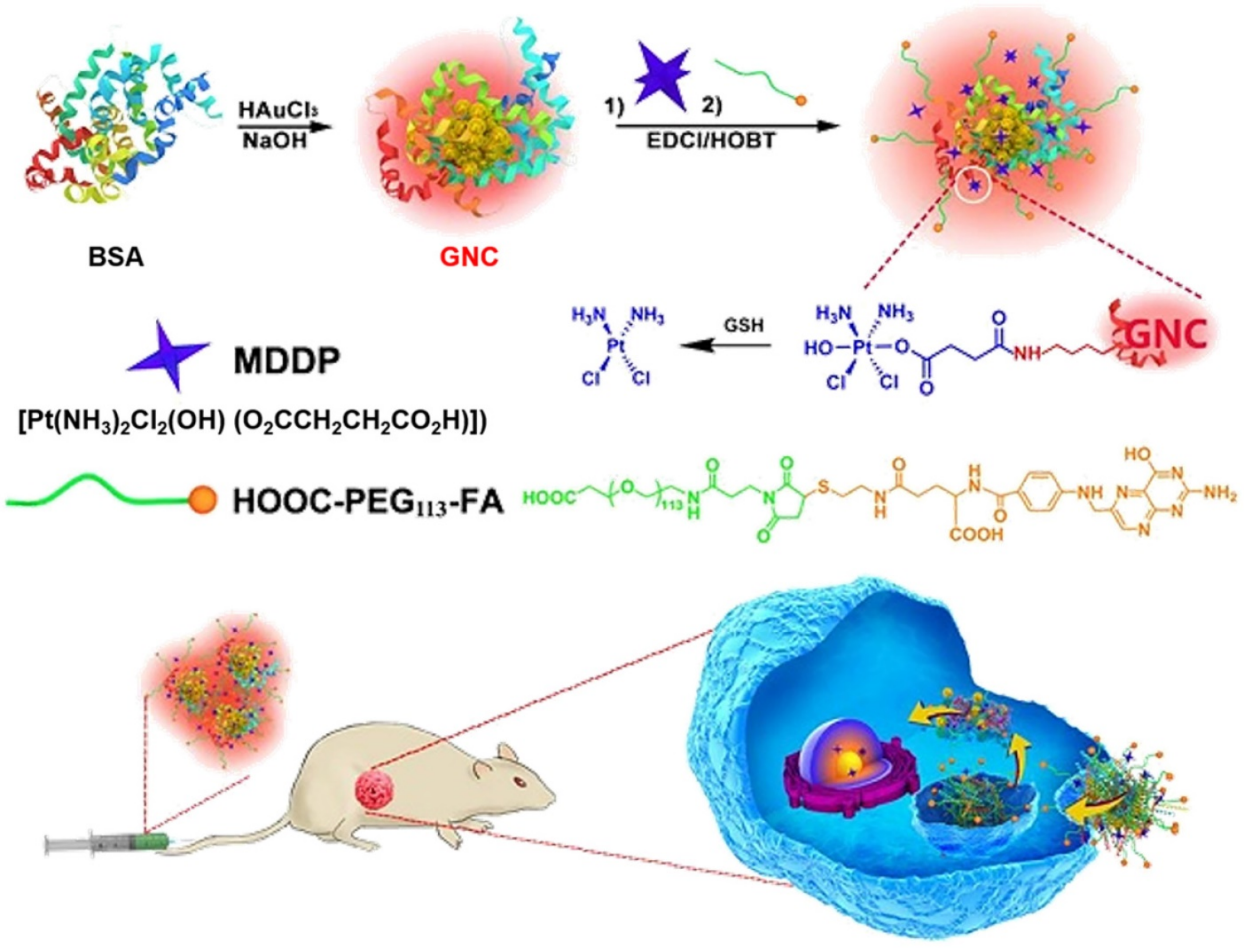
Schematic illustration of GNC-based theranostic nanoplatform for tumor-targeted chemotherapy and fluorescence imaging. Adapted with permission from ref 18, copyright 2016 Ivyspring International Publisher.

Utilizing the unique biological effects of GNCs, Zhou et al. employed BSA-protected GNCs as a dual-functional nanoplatform for drug delivery and fluorescence imaging of the tumor (Figure 3) 18. The GNCs were first conjugated with reduction-sensitive cisplatin coupled with a prodrug (cis,cis,trans[Pt(NH_3_)_2_Cl_2_(OH)(O_2_CCH_2_CH_2_CO_2_H)]) (MDDP), and then functionalized with a targeting ligand folic acid (FA) 92, which can target folate receptor α (FR-α) overexpressed on the surface of cancer cells 93. Using the highly aggressive 4T1 breast cancer cell line and its orthotopic tumor model, the investigators demonstrated selective accumulation of the prodrug and FA dual-conjugated GNCs inside the cancer cells and the tumor. The nanoparticles could efficiently inhibit the growth of the primary tumor and suppress metastasis of cancer cells to the lung. These data demonstrated the good potential of the GNC-based theranostic nanoplatform for fluorescence tumor imaging and cancer therapy and the advantages of GNCs as a drug delivery platform. First, when BSA is used for nanoparticle synthesis, GNCs are biocompatible and biodegradable. For clinical translation, BSA can be replaced with human serum protein (HSA) without changing the physicochemical properties of the nanoparticles. Second, the prodrug and FA dual-conjugated GNC nanoparticles selectively accumulated in the orthotopic 4T1 tumor model, displayed high fluorescence signals for nanoparticle tracking and tumor imaging 94 and efficiently inhibited primary tumor growth and suppressed metastasis of cancer cells to the lung. Moreover, the hydrodynamic diameter of FA-GNC-Pt was ~10 nanometers, allowing escape from the reticuloendothelial system (RES) in the liver and fast kidney clearance, avoiding liver accumulation and minimizing side effects.

**Figure 4:**
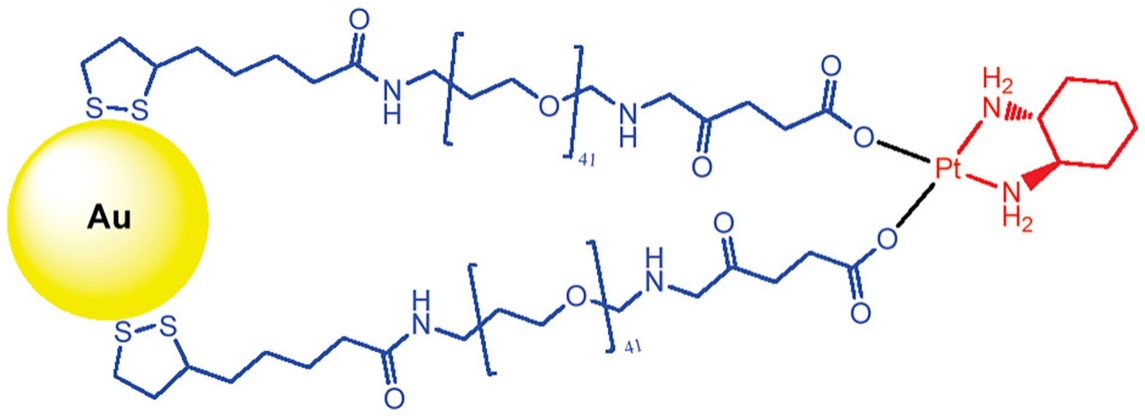
Chemical synthesis of the platinum-tethered gold nanoparticles. Adapted with permission from ref 95, copyright 2010 American Chemical Society.

Brown et al. tethered the active component of the anti-cancer drug oxaliplatin to gold nanoparticles (AuNPs) for improved drug delivery 95. Poly(ethylene glycol) (PEG)-modified AuNPs have been used to functionalize cisplatin or oxaliplatin. For example, the active component of oxaliplatin was tethered to AuNPs that were functionalized with a thiolated PEG monolayer capped with a carboxylate group. The platinum-tethered NPs demonstrated comparable or significantly higher cytotoxicity than oxaliplatin against the A549 epithelial lung cancer and several colon cancer cell lines. In particular, the nanoparticles showed an unusual ability to penetrate the nucleus in lung cancer cells. The platinum-tethered nanoparticles demonstrated as good as, or significantly better, cytotoxicity than oxaliplatin alone in all cell lines (HCT116, HCT15, HT29, and RKO) and an unusual ability to penetrate the nucleus in lung cancer cells (Figure 4).

#### Magnetic Iron Oxide Nanoparticles as Drug Carriers

Superparamagnetic iron oxide nanoparticles (SPIONs) are usually employed as targeted delivery regents due to their advantages of low toxicity, biocompatibility, biodegradability, and well water dispersion. SPIONs can bind to drugs and be directed to the tissues of interest or tumors using an external magnetic field 96, 97.

**Figure 5:**
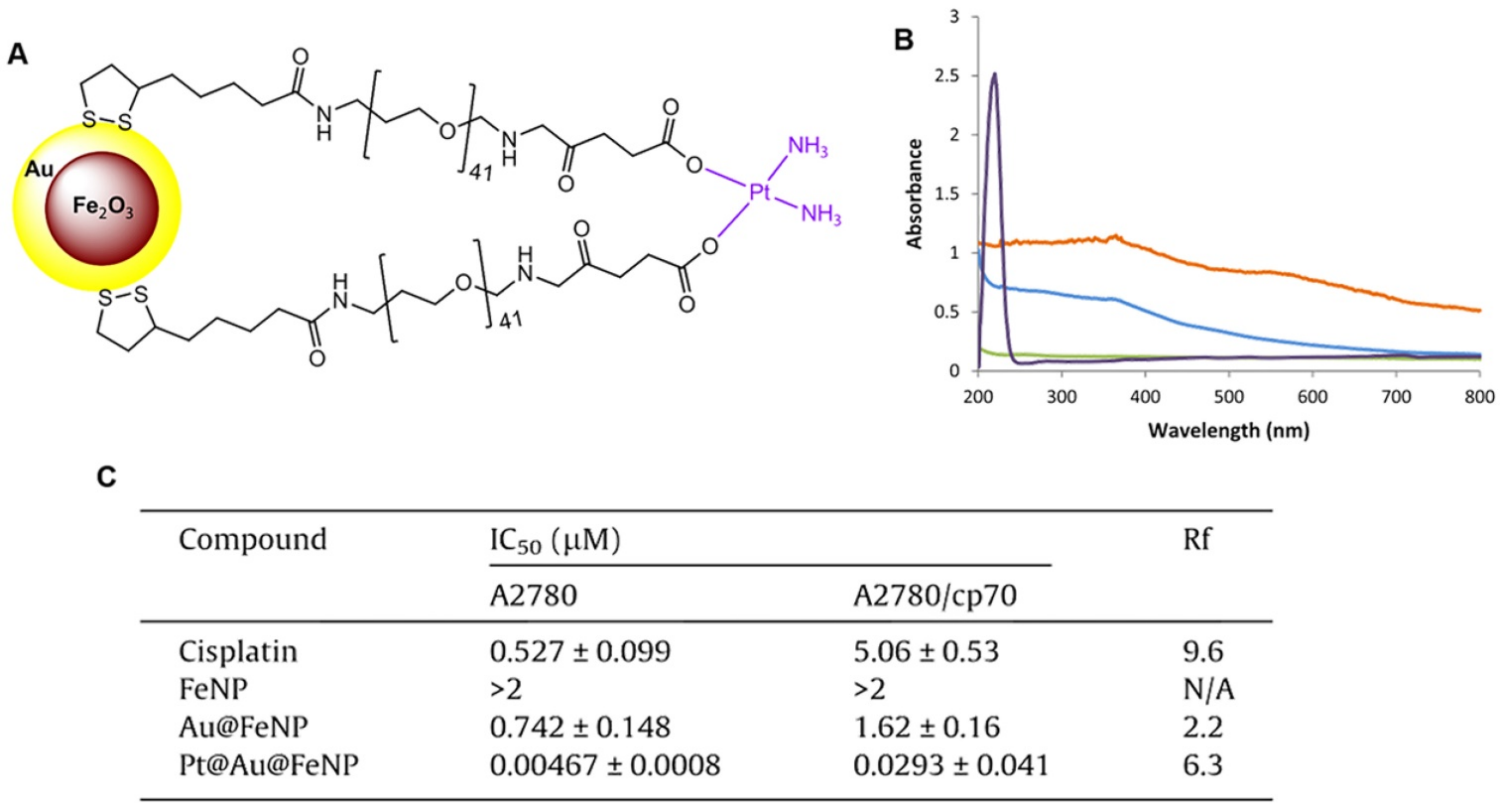
** (A)** Delivery system of gold-coated iron oxide nanoparticles functionalized with thiolated polyethylene glycol (PEG) linkers to which the active component of the anti-cancer drug cisplatin, [Pt(NH_3_)_2_]^2+^, is attached via the terminal carboxylate groups. **(B)** UV-Vis spectra of the four nanoparticles: FeNPs (blue), Au@FeNPs (orange), PEGylated Au@FeNPs (green), and Pt@Au@FeNPs (purple). **(C)** *In vitro* cytotoxicity of the nanoparticles in the human ovarian carcinoma cell line A2780 and its cisplatin-resistant sub-line A2780/cp70. Resistance factor (Rf) is defined as the IC_50_ of the complex in the resistant line divided by the IC_50_ of the complex in the sensitive line; any complex with an Rf less than 1 can overcome cisplatin resistance. Adapted with permission from ref 98, copyright 2012 Elsevier.

Wagstaff et al. tethered the active component of cisplatin to gold-coated iron oxide nanoparticles (Pt@Au@FeNPs) to improve their delivery to tumors and increase efficacy 98. The nanoparticle-based drug delivery system of Pt@Au@FeNPs was functionalized with thiolated polyethylene glycol (PEG) linkers to which the active component of the anti-cancer drug cisplatin, [Pt(NH_3_)_2_]^2+^ was attached via the terminal carboxylate groups (Figure 5A). The successful introduction of cisplatin was confirmed by UV-visible spectra (Figure 5B). The cytotoxicity of the Pt@Au@FeNPs was examined using *in vitro* growth inhibition assays in the human ovarian carcinoma cell line A2780 and its cisplatin-resistant derivative A2780/cp70. Pt@Au@FeNPs demonstrated activity at nanomolar concentrations and were 110-fold more active than cisplatin in A2780 cells, while iron oxide nanoparticles (FeNP) showed no cytotoxicity at concentrations up to 2 μM. However, in this study, the cisplatin Pt@Au@FeNPs, despite having activity at nanomolar concentrations, were cross-resistant with cisplatin in A2780/cp70 cells (Figure 5C).

**Figure 6:**
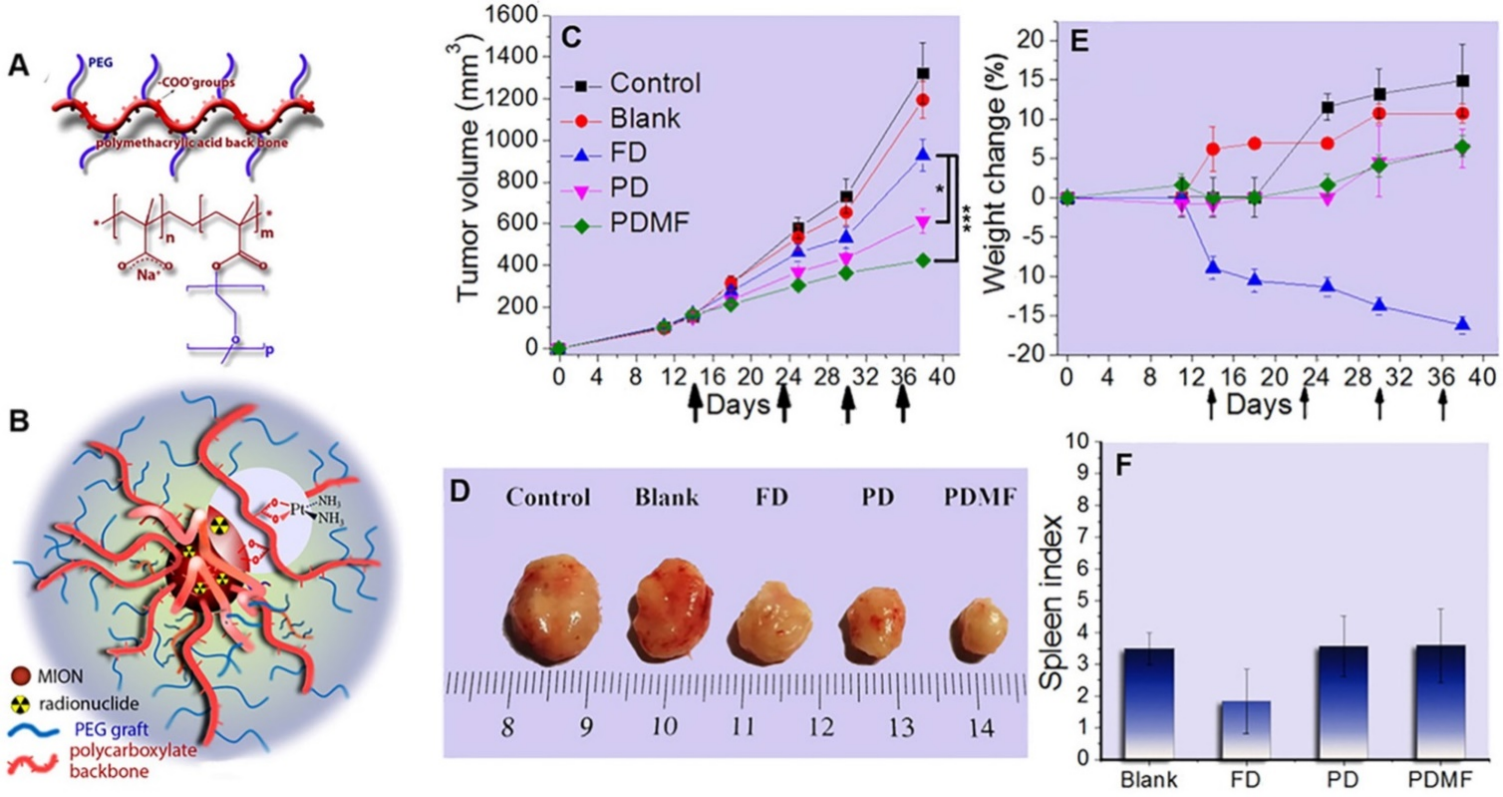
** (A)** Chemical structure of copolymers poly(methacrylic acid)-graft-poly(ethyleneglycol methacrylate) (p(MAA-g-EGMA)). **(B)** Schematic structure of the studied magnetic drug delivery systems. Evolution with time of **(C)** Tumor volume and (E)% weight change of mice (n=4) after i.v. injections with: saline (Control), nanocarriers without the drug (Blank), aqueous cisplatin solution (FD), cisplatin-loaded nanocarriers (PD), and cisplatin-loaded nanocarriers in the presence of an external magnetic field in the tumor area (PDMF). Arrows in (a) and (b) represent tail vein injection events. **(D)** Pictures of the tumors taken at the end of the study period are shown for comparison. **(F)** Spleen index of mice sacrificed at the end of the *in vivo* experiment. Adapted with permission from ref 99, copyright 2016 Elsevier.

SPIONs are promising drug carriers because of the targeted delivery to tumors and increased efficacy through external magnets. Voulgari et al. synthesized a magnetic nanocarrier poly(methacrylic acid)-graft-poly(ethyleneglycol methacrylate) (p(MAA-g-EGMA)) by radical copolymerization of methoxy-PEG-methacrylate with methacrylic acid (Figure 6A). The cisplatin-loaded (PD) magnetic nanocarriers (Figure 6B) facilitated magnetically-triggered drug release and displayed *in vitro* anti-cancer activity comparable to free cisplatin at the same drug concentrations. In addition, they exhibited an enhanced anti-cancer effect *in vivo* on a cisplatin-resistant HT-29 tumor model in mice, particularly when a magnetic field was applied to the tumor area (Figure 6C-D). During the study period, a decrease in mouse weight was observed in the free cisplatin group but not in the p(MAA-g-EGMA)-treated group (Figure 6E), indicating a significant reduction of side-effects with the cisplatin-loaded magnetic nanocarriers. Moreover, spleen indices in the groups of mice injected with cisplatin-loaded magnetic nanocarriers (Figure 6F) were identical with the control group, suggesting a pronounced reduction of cisplatin systemic toxicity 99.

#### Other Nanomaterials as Drug Carriers

Besides SPIONs and GNCs, other nanomaterials, such as mesoporous silica, have multiple potential applications as drug carriers 100, 101. Mesoporous silica nanoparticles (MSNs) with large surface area and pore volume have an extraordinary ability to store drugs, and the controllable release of Pt drugs from the designed mesoporous structures is advantageous for the bioavailability of drugs 102. Also, organic nanoparticles, such as polymeric nanoparticles, exhibit great potential in drug delivery because of their unusual properties, including simple encapsulation, high capacity, controlled release, and low toxicity. The benefits of encapsulating Pt drugs in polymeric nanoparticles to reduce side effects without affecting drug efficacy have been demonstrated in tumor-bearing mice and preclinical cancer models 103.

### Glutathione-Scavenging Pt Drugs

Glutathione (GSH) is one of the most abundant non-protein thiols in tumor cells, with its intracellular content of about 0.5-10 mM 37, 104, 105 and is the most important intracellular thiol compound which participates in cellular detoxification mechanisms 106, 107. Previous reports indicated that cancer cells could utilize endogenous GSH to chelate Pt drugs and produce inactive GSH-Pt adducts, which can be preferentially pumped out via membrane transport proteins and are non-toxic to cancer cells 12, 108, 109. In this context, GSH-scavenging Pt drugs have been reported.

**Figure 7:**
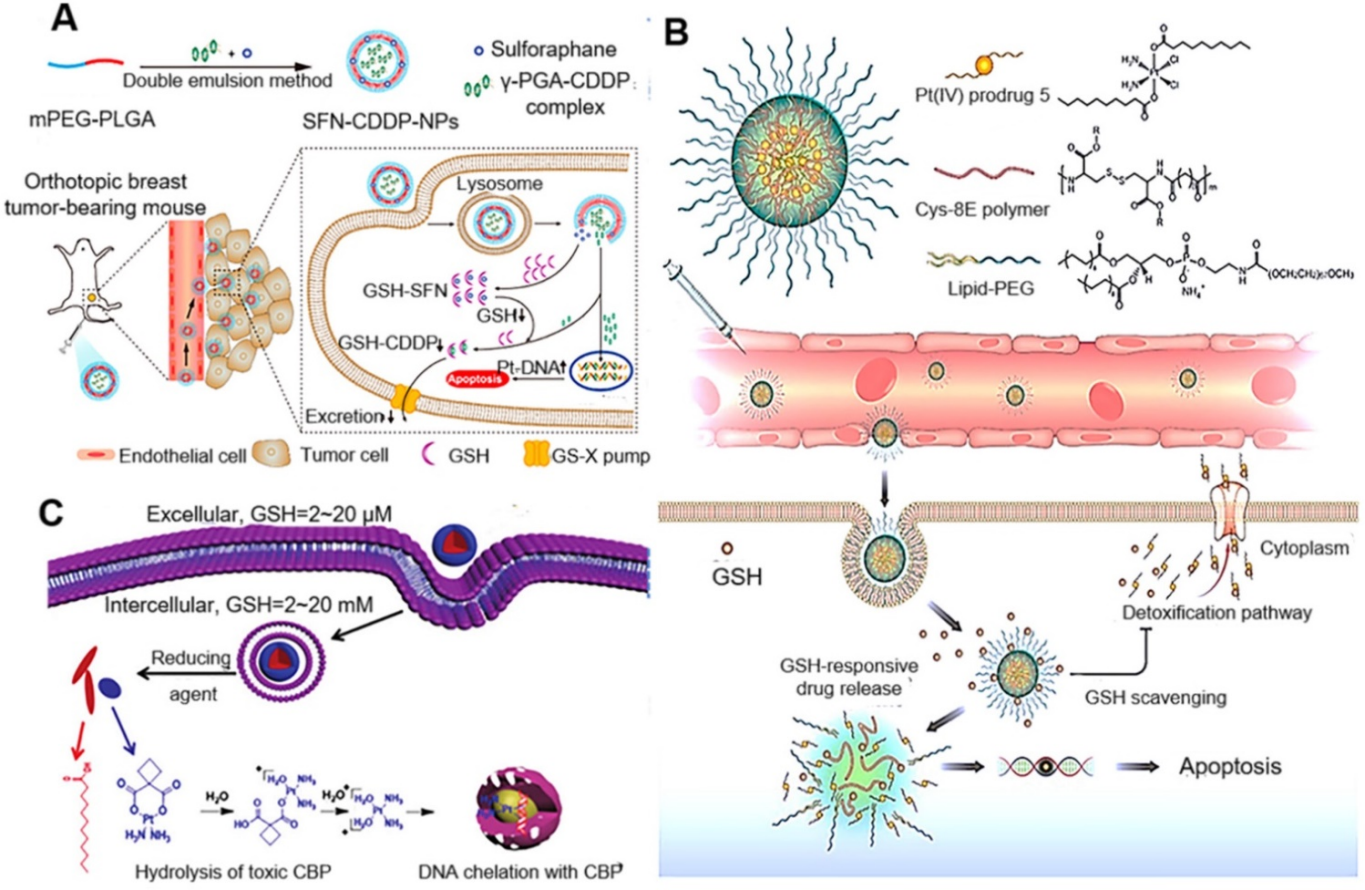
** (A)** Schematic diagram of the preparation of SFN-CDDP-NPs for improved anti-tumor therapy. Adapted with permission from ref 110, copyright 2020 American Chemical Society. **(B)** Illustration of the redox-responsive nanoplatform, composed of Pt(IV) prodrug 5, Cys-8E polymer, and lipid-PEG, for *in vivo* Pt delivery and treatment of cisplatin-resistant tumors. Adapted with permission from ref 111, copyright 2018 American Chemical Society. **(C)** Schematic illustration of phospholipid-mimic CBP-LA conjugates that self-assemble into micelle-like nanoparticles and the possible mechanism of their anti-cancer activity. Adapted with permission from ref 112, copyright 2018 Royal Society of Chemistry.

Sulforaphane (SFN) has been reported to deplete GSH by directly binding with GSH to form the GSH-SFN complex, which can be exported out of the cell. Recently, Xu et al. proposed that the therapeutic efficacy of SFN-CDDP-NPs could be significantly improved by SFN-mediated GSH depletion 110. As displayed in Figure 7A, the investigators designed an NP-enabled codelivery system consisting of a water-soluble poly(γ, L-glutamic acid)-CDDP (γ-PGA-CDDP) conjugate and SFN for breast cancer treatment. The therapeutic efficacy of SFN-CDDP-NPs was systematically investigated and compared with free drugs both *in vitro* and *in vivo*. After efficient internalization of SFN-CDDP-NPs by tumor cells, the rapidly released SFN could notably decrease the GSH content and thus significantly increase DNA-bound Pt, resulting in severe DNA damage and cellular apoptosis. Due to the improved chemosensitivity and preferential tumor accumulation, SFN-CDDP-NPs greatly inhibited orthotopic breast cancer progression with reduced toxic side effects.

Ling et al. reported the synthesis of cysteine-based poly(disulfide amide) (Cys-PDSA) polymers (Cys-8E polymer) that readily react with GSH via disulfide-mediated reduction and their combination with a series of Pt(IV) prodrugs with tunable hydrophobicity. Optimized polymers rapidly disassembled and released Pt drugs in response to intracellular GSH while simultaneously consuming GSH to restore Pt sensitivity in cisplatin-resistant tumor cells 111. Moreover, *in vivo* efficacy and safety results showed that NPs effectively inhibited the growth of cisplatin-resistant xenograft tumors with an inhibition rate of 83.32% while alleviating serious side effects associated with cisplatin. GSH-scavenging polymeric NP technology reported herein could provide a unique strategy for improving the therapeutic efficacy of current Pt drugs (Figure 7B). Liang et al. also developed novel small-molecule-based nanodrugs of carboplatin-lauric acid nanoparticles (CBP-LA NPs) to reduce GSH-mediated platinum resistance and improve the anti-tumor efficiency of Pt(II) 112. The intracellular glutathione determination and the Pt-DNA adduct assay revealed that CBP-LA NPs could reduce intracellular GSH levels and improve the efficiency of platinum chelating with DNA to overcome GSH-mediated Pt(II) resistance (Figure 7C).

## Pt Nanoclusters (Pt NCs) as a New Pt Drug for Cancer Therapy

### Properties of Pt Nanoclusters

Pt NCs, similar to noble metal clusters such as GNCs, are relatively stable molecular aggregates composed of up to hundreds of metal atoms 113. Their physical size is normally between atoms and nanoparticles, close to the Fermi wavelength of a single electron. Pt NCs have attracted much attention in bioanalysis and biomedicine due to their special physicochemical properties, such as ultra-small size, precise structure, photoluminescence, X-ray absorption, low cytotoxicity, and good biocompatibility 114, 115. In particular, due to distinct molecular composition and a good biological safety profile, Pt NCs have great application prospects in anti-cancer treatment.

### Application of Pt NC-based Drugs in Cancer Therapy

Although multiple studies of the Pt(II) complex and modified Pt(IV) prodrugs have attempted to improve the anti-cancer efficiency of Pt drugs, these strategies relied on anti-cancer mechanisms similar to cisplatin and were not very successful 116-118. In this section, we discuss Pt NCs as anti-cancer drugs with different mechanisms for cancer therapy.

**Figure 8:**
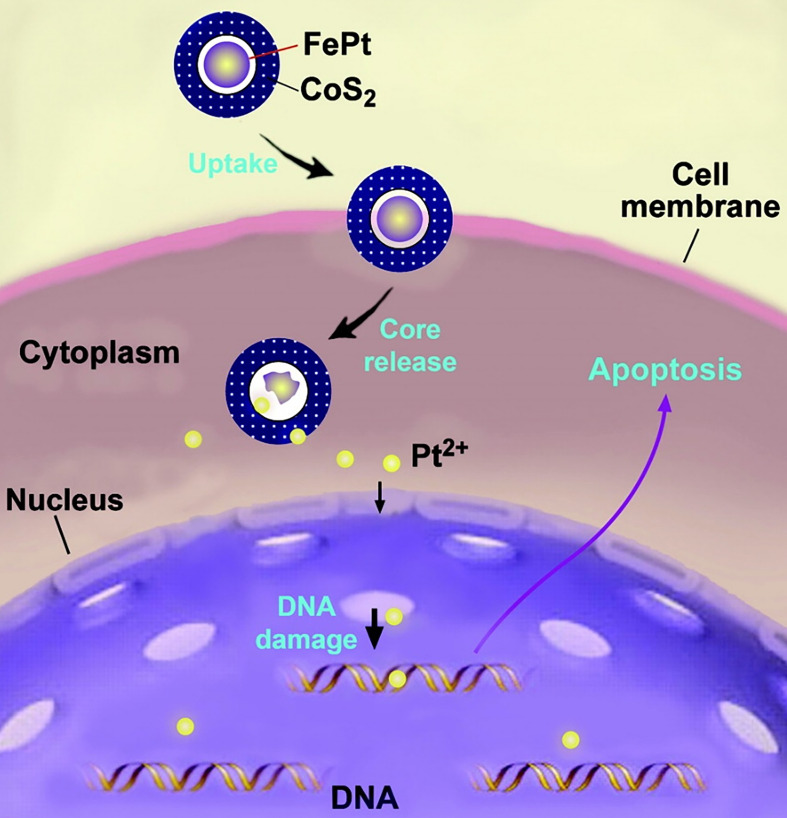
Illustration of a possible mechanism accounting for FePt@CoS_2_ yolk-shell nanocrystals killing HeLa cells. After cellular uptake, FePt nanoparticles were oxidized to generate Fe^3+^ (omitted for clarity) and Pt^2+^ ions (yellow). The Pt^2+^ ions enter into the nucleus (and mitochondria), bind to DNA, and lead to apoptosis of the HeLa cell. Adapted with permission from ref 120, copyright 2008 American Chemical Society.

So far, the cytotoxicity mechanism of Pt NCs is still unclear because of the differences in size, shape, surface coatings, and purity of the particles 119. Nevertheless, it is generally accepted that the cytotoxicity of Pt NCs depends primarily on abundant Pt^2+^ ions leaching under low pH conditions, such as in cell endosomes, to induce DNA damage 32. As early as 2007, the inhibitory mechanism of Pt NCs on tumor cells was described. Gao et al. reported that the synthesized FePt@CoS_2_ yolk-shell nanoclusters exhibited an IC_50_ of 35.5 ng/mL (4.7 ng/mL of Pt) in HeLa cells that was much lower than cisplatin (230 ng/mL of Pt). The FePt nanoclusters were oxidized to generate Pt^2+^ and Fe^3+^ ions, especially in intracellular late lysosome with a low pH (pH < 5.5) environment, as hollow nanospheres were found in mitochondria of cancer cells, implying breakdown of the FePt core. Thus, after cellular uptake, FePt cores disintegrated to generate metal ions inside the acidic environment of secondary lysosomes. Subsequently, these metal ions could escape from endosomes and enter the cell nucleus to bind DNA, forming DNA-Pt adducts and eventually leading to tumor cell apoptosis (Figure 8). Furthermore, transmission electron microscopy (TEM) confirmed cellular uptake of FePt@CoS_2_ nanocrystals, and the magnetic properties analysis corroborated the release of FePt nanoparticles from yolk-shell nanostructures after cellular uptake 120.

**Figure 9:**
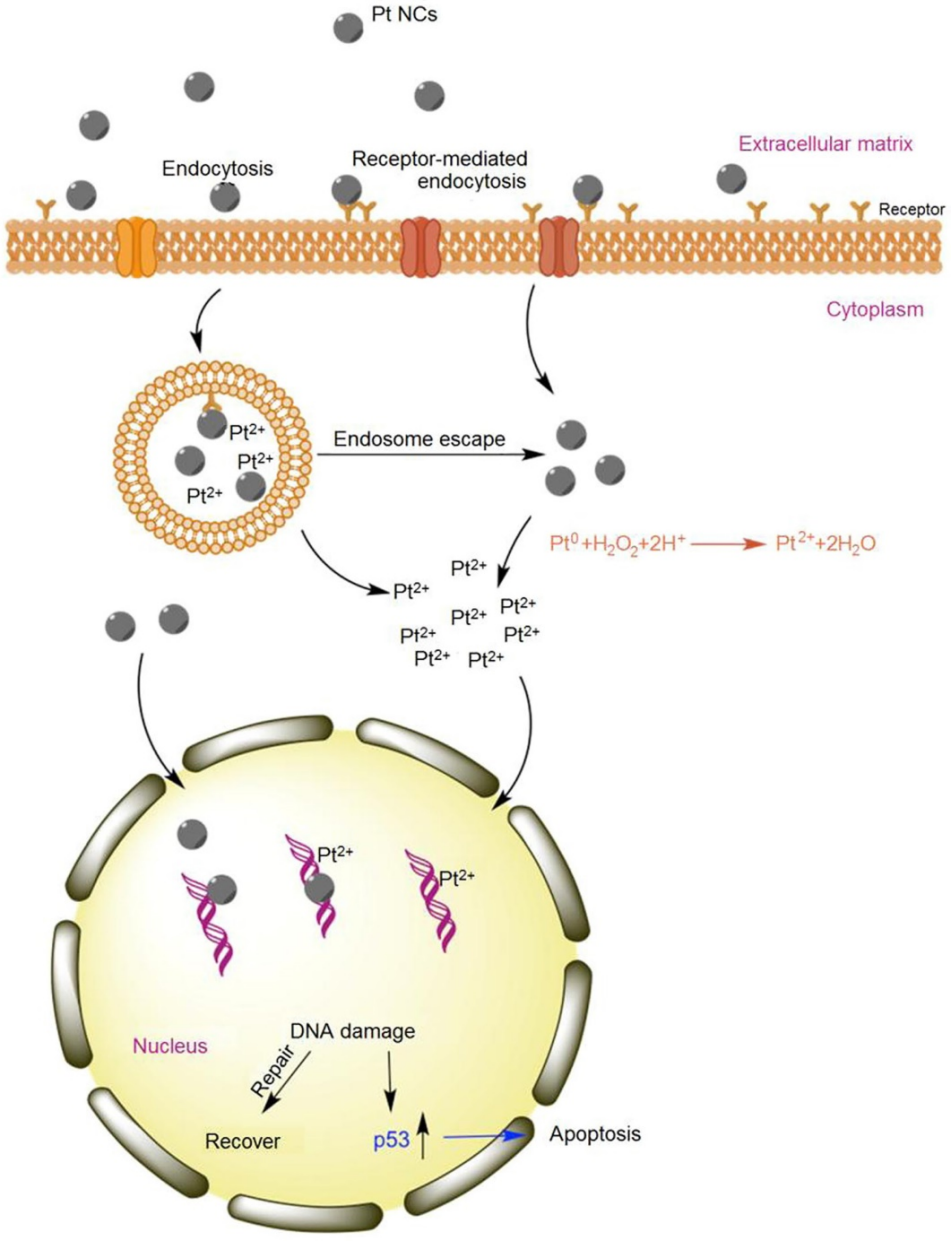
Schematic of apoptosis mechanism of Pt NCs. Abundant oxidized Pt ions and Pt NCs coordinate the DNA damage activating the p53 pathway. Adapted with permission from ref 32, copyright 2017 Elsevier.

Although the anti-tumor mechanism of Pt NCs remains obscure, it is believed that most Pt atoms are exposed on the ultrasmall <2 nm size nanocluster surface 36. These high surface-active Pt NCs are affected by intracellular acidic organelles like endosomes and lysosomes and then rapidly decompose to form oxidative states of Pt (Figure 9) that can attach to and change DNA structure, resulting in cancer cell apoptosis. In addition, ultrasmall Pt NCs can anchor onto the grooves of DNA double helix to further damage the DNA. Thus, eradicating cancer cells by Pt NC-based nanodrugs appears to be the synergistic effect of both Pt NCs and Pt ions causing DNA damage 32.

**Figure 10:**
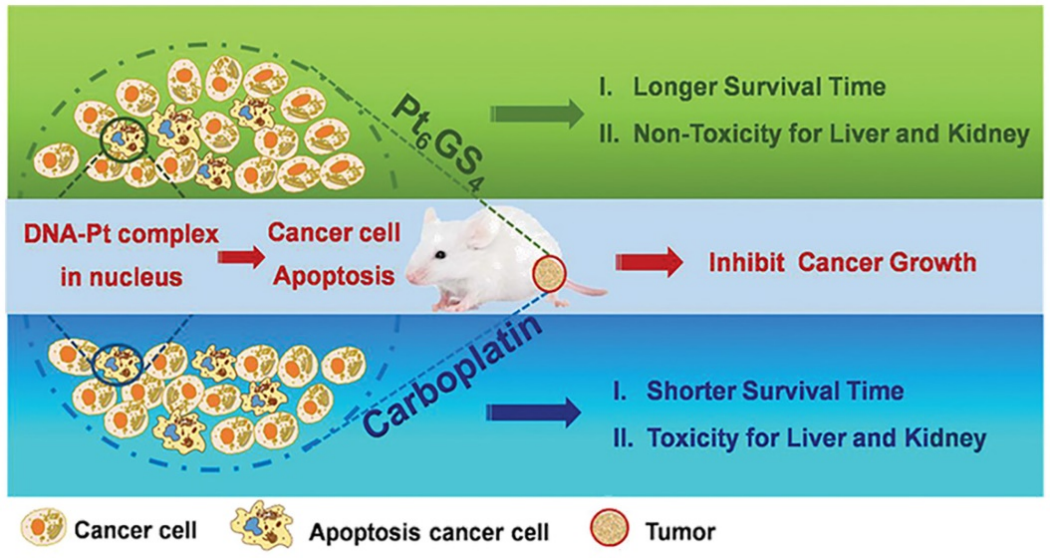
Schematic illustration of GSH-chelated Pt molecule (Pt_6_GS_4_) as a potent anti-cancer agent. High efficacy for anti-cancer treatment and lower systemic toxicity were achieved by Pt_6_GS_4_ both *in vitro* and *in vivo*, compared to carboplatin at the same dosage. Adapted with permission from ref 121, copyright 2020 Wiley.

Recently, our research group explored a facile approach to develop an endogenous GSH-chelated Pt molecule containing multiple Pt atoms for efficient cancer treatment (Figure 10) 121. These polynuclear Pt NCs were identified by electrospray ionization mass spectra (ESI-MS) and density functional theory (DFT) study as Pt_6_GS_4_. High efficacy for anti-cancer treatment was achieved by Pt_6_GS_4_ both *in vitro* and *in vivo* when compared with traditional first-line carboplatin at the same dosage. The Pt_6_GS_4_ molecule could be readily taken up by aggressive triple-negative breast cancer (TNBC) cells. Subsequently, its metabolites entered nuclei to interact with DNA, and finally, the DNA-Pt complex triggered TNBC cell apoptosis via the p53 pathway. These data revealed that Pt_6_GS_4_ was comparable to carboplatin for cancer cell uptake, nuclear localization, and cancer cell proliferation inhibition. More significantly, compared with carboplatin, Pt_6_GS_4_ was non-toxic for the liver and kidneys, and Pt_6_GS_4_-treated mice lived longer. Our study opened a new avenue to explore polynuclear Pt compounds with accurate architecture for enhancing therapeutic effects and reducing systemic toxicity.

Targeting peptides have widely been used to synthesize Pt NCs 24, 113, 122, 123, improving the existing first-line platinum drugs in the clinic that inhibit rapid proliferation of tumor cells, and can also improve drug bioavailability. For example, Feng et al. synthesized mitochondria-targeting Pt NCs (CytcApt-Pt NCs) using cytochrome c aptamer (CytcApt) as a template. *In vitro* experiments showed that CytcApt-Pt NCs could kill 4T1 tumor cells in a pH-dependent manner but did not affect normal 293T cells. These results showed good therapeutic efficacy and excellent biosafety of CytcApt-Pt NCs, indicating their great potential for tumor treatment and reducing systemic toxicity 124.

**Figure 11:**
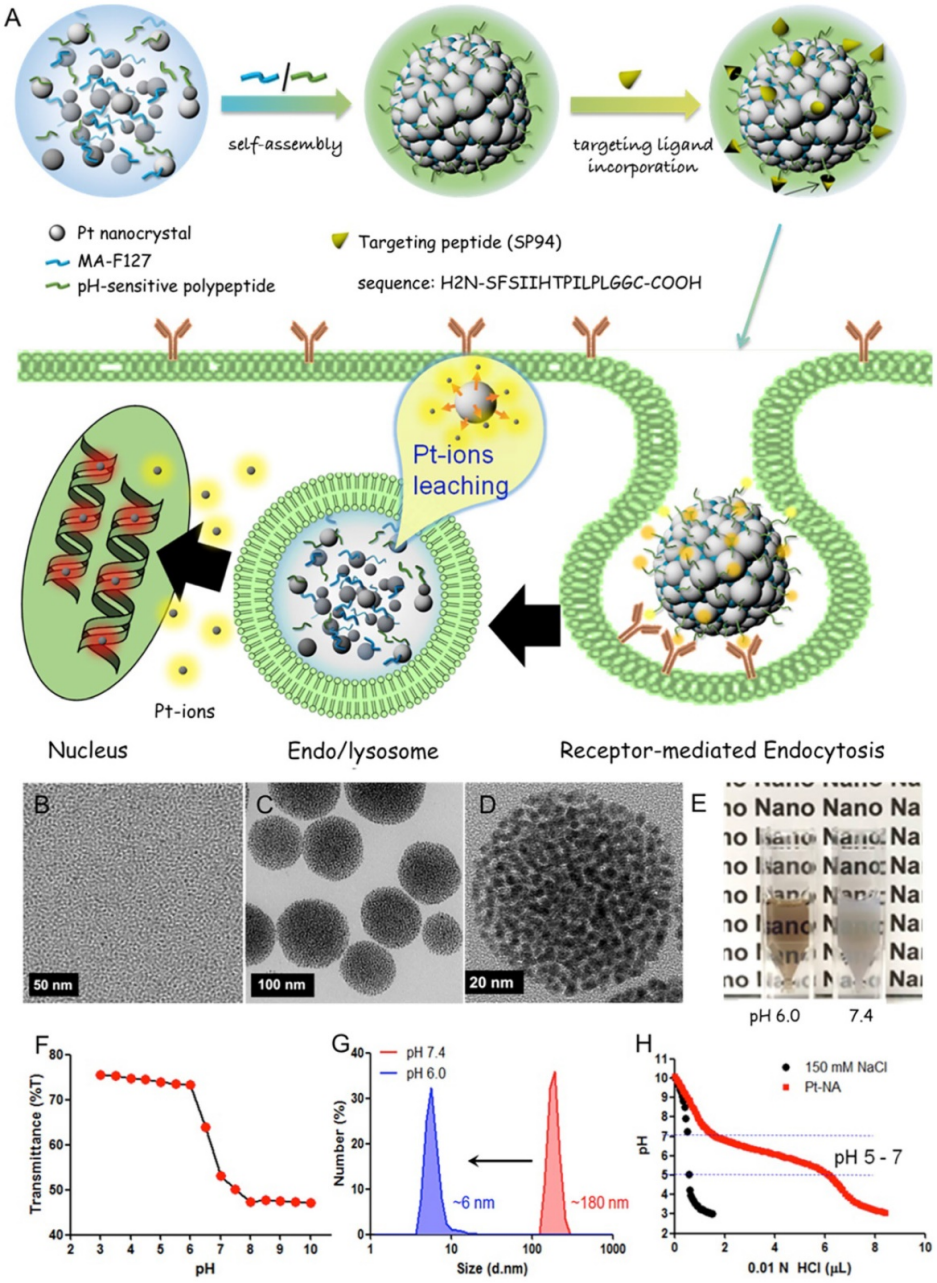
Design and characterization of HCC-targeted pH-sensitive Pt nanocluster assembly (Pt-NA). **(A)** Schematic representation of Pt-NA synthesis, targeted HCC uptake, and intracellular Pt ion release. **(B)** TEM image of the synthesized Pt NCs. **(C)** TEM image of Pt-NA. **(D)** High-resolution TEM image of Pt-NA. **(E)** Photographs of Pt-NA in pH 6.0 and 7.4. **(F)** The transmittance of a suspension of Pt-NA as a function of pH. **(G)** DLS size measurement of Pt-NA (0.1 mg mL^-1^) as a function of pH. **(H)** pH profile of Pt-NA by acid-base titration. Adapted with permission from ref 125, copyright 2016 American Chemical Society.

Xia et al. synthesized a Pt nanocluster assembly (Pt-NA) composed of assembled Pt NCs incorporating a pH-sensitive polymer and hepatocellular carcinoma (HCC)-targeting peptide (Figure 11) 125. Pt-NA was latent in peripheral blood, readily targeted disseminated HCC cancer stem-like cells (CSLCs), and disassembled into small Pt NCs in acidic subcellular compartments, eventually inducing DNA damage. Moreover, the study demonstrated the underlying mechanism of these effects at the molecular level as downregulation of many genes that are highly expressed in liver cancer patients. Thus, Pt-NA has a good potential in clinical HCC treatment 125.

**Figure 12:**
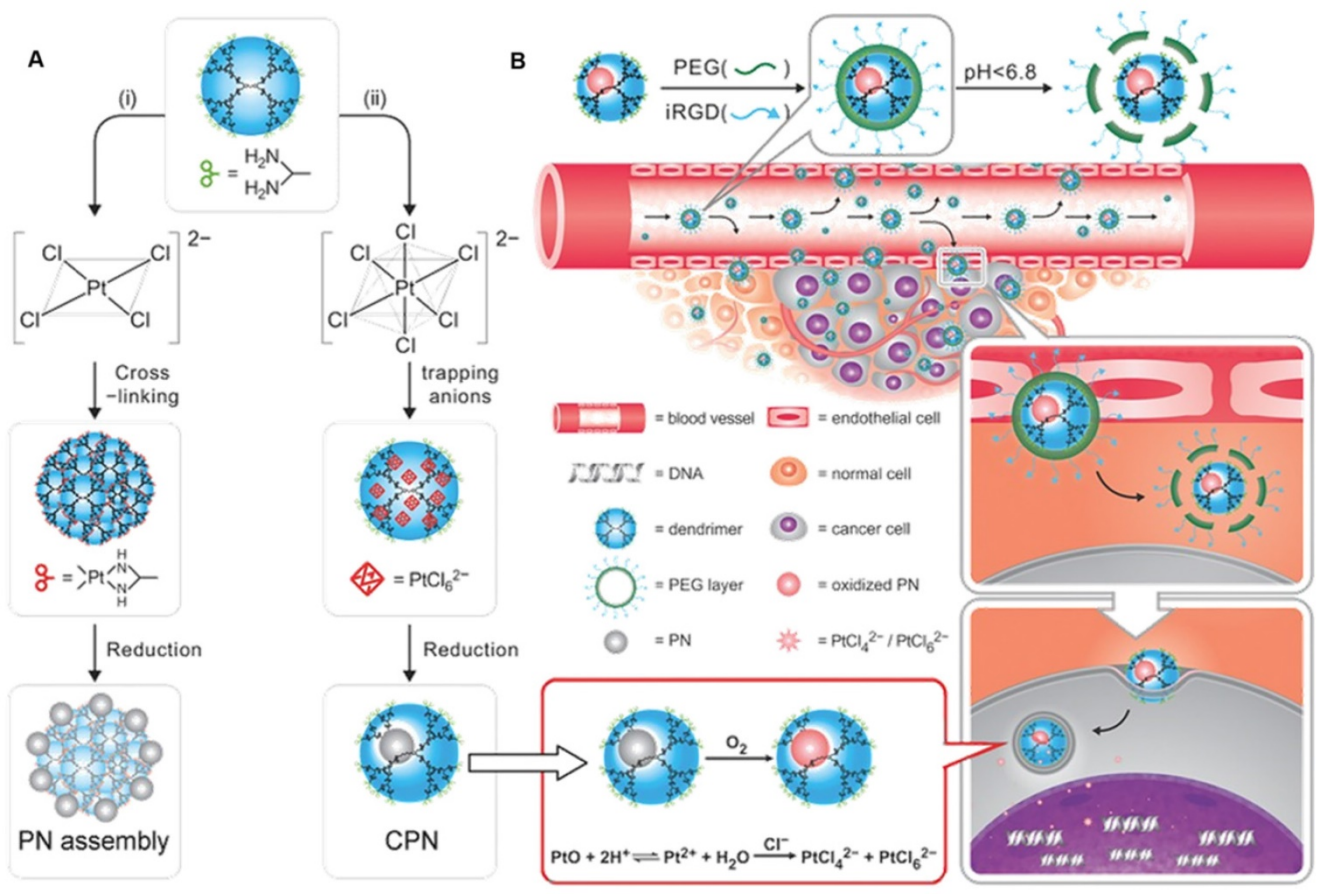
** (A)** Schematic representation of a novel strategy based on tuning anionic geometry for the formation of PN. **(B)** Schematic representation of the caged PN mixed with a tumor-penetrating peptide to target the tumor and kill malignant cells by shedding the outer PEG corona to exert tumor-inside activation. Adapted with permission from ref 126, copyright 2013 Wiley.

Another example is of a first-generation dendrimer-caged Pt nanocluster (CPN) with the size of an atomic level (0.93 ± 0.22 nm in diameter). CPN was endowed with targeting function by conjugating with the iRGD peptide (Figure 12A) 126. Especially, CPN could be easily oxidized, resulting in the loss of its intrinsic chemical inertness and its surface corrodibility for further dissolution in weakly acidic organelles, such as endosomes and lysosomes, to release toxic Pt ions for DNA cross-linking (Figure 12B). Employing subcutaneous breast cancer xenografts in mice, the therapeutic effect of CPN was examined by intratumoral injection *in vivo.* Results indicated that this chemotherapeutic had efficacy comparable to cisplatin.

## Conclusion and Perspectives

Traditional tumor chemotherapy employs chemo-drugs, which usually have strong side effects for normal cells and tissues 13. Pt-based anti-cancer drugs play a vital role in clinical cancer therapy with satisfactory efficacy. The first-line clinical platinum anti-cancer drugs represented by cisplatin are relatively old drugs that have a therapeutic effect on tumors with a known molecular mechanism. However, the side effects seriously limit the application of platinum anti-cancer drugs. Therefore, modified Pt-based drugs, which could improve anti-cancer efficiency and reduce systemic toxicity have been investigated. Pt NC-based nanodrugs have attracted much attention due to the inherent higher blood circulation time, EPR effect, and facile surface functionalization. Advances in nanotechnology and nanoscience have facilitated the development of Pt NCs, representing an important research orientation for exploring platinum drugs with the precise structure to improve therapeutic effect and reduce systemic toxicity 127.

The challenges accompanying these advances provide us with future directions and efforts for designing and constructing more effective Pt-based drugs for possible clinical applications:

(1) For cisplatin and other platinum anti-cancer drugs, systemic toxicity is still the most challenging problem. Platinum drugs are modified by many methods, such as linking target molecules and adding drug delivery carriers, with the ultimate goal of reducing their toxicity.

(2) Many new platinum nanodrugs, such as Pt NCs, have been developed. Pt NCs generate platinum ions in cells and induce irreversible DNA damage 32, 37, 125, 126. However, Pt NC-based drugs are still cytotoxic, and the possible harmful mechanisms are not entirely understood. Investigations on the role of Pt NCs size in cytotoxicity indicated that it could represent an important parameter affecting molecular mechanisms. Recent synchrotron radiation X-ray techniques may provide insights into nanomaterial biotransformation to address the anti-tumor mechanism of Pt NCs. It is crucial to study the dynamic process of Pt NCs metabolism *in vivo* and their interaction with biomolecules to treat malignant tumors and other diseases 128, 129, which would be critical for designing Pt NC-based nanodrugs and high efficiency.

(3) As a new type of platinum anti-cancer drug, the molecular composition of Pt NCs needs to be further improved. Mass spectrometric techniques, such as MALDI-TOF-MS and ESI-MS, would help characterize the molecular formula of clusters or the metal to ligand ratio in clusters, but the precise structural characterization methods of cluster molecules need to be further explored 130-133. We believe that nanotechnology would be immensely helpful in addressing these issues. Besides understanding the precise molecular composition of Pt NCs, we need to study the impact of Pt NCs on their anti-tumor function in the context of the configuration of cisplatin and transplatin, which is an area worthy of in-depth exploration. The application of nanotechnology in the field of Pt NC-based nanodrugs undoubtedly provides a promising prospect for improving the current anti-cancer treatment.

(4) Combining Pt-based nanodrugs with other therapeutic methods, such as synergistic chemo-electrodynamic therapy, can maximize the bio-function of Pt NCs and strengthen their anti-tumor effect 102, 134-136. In addition to individual Pt-based drugs, bimetallic composites, such as platinum complexes in combination with ruthenium, also showed excellent anti-cancer performance 57, 137. This represents a new strategy to overcome the deactivation pathways during Pt drug treatment and a good option as a promising anti-cancer agent.

## Figures and Tables

**Table 1:** Timeline of major milestones in three generations of first-line Pt drug clinical application

Generation	Pt Drug	Molecular Structure	Market Time	Listed Country
First	Cisplatin	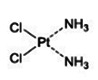	1978	Japan/Italy
Second	Carboplatin	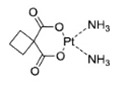	1986	America
Third	Oxaliplatin	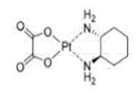	1996	France

## Abbreviations

CDDP: cisplatin
CTR1: copper transporter 1
ER: endoplasmic reticulum
RGD: arginine-glycine-aspartic
AIE: aggregation-induced emission
CPP: cell-penetrating peptides
TPP: tumor penetrating peptide
LHRH: luteinizing hormone-releasing hormone
GNC: gold nanoclusters
Pt NCs: platinum nanoclusters
MSNs): mesoporous silica nanoparticles
SPIONs): superparamagnetic iron oxide nanoparticles
FA: folic acid
HSA: human serum protein
RES: reticuloendothelial system
AuNPs: gold nanoparticle
PEG: polyethylene glycol
GSH: Glutathione
SFN: Sulforaphane
CBP-LA NPs: carboplatin-lauric acid nanoparticles
ESI-MS: electrospray ionization mass spectra
DFT: density functional theory
TNBC: triple-negative breast cancer
BSA: bovine serum albumin protein
HCC: hepatocellular carcinoma
CPN: dendrimer-caged Pt nanoclusters
PN: Pt nanoclusters
Pt-NA: Pt nanocluster assembly
MALDI-TOF-MS: matrix-assisted laser desorption/ionization-time of flight mass spectrometry
